# The Impact of Highly Pathogenic Avian Influenza H5N1 in the United States: A Scoping Review of Past Detections and Present Outbreaks

**DOI:** 10.3390/v17030307

**Published:** 2025-02-24

**Authors:** Alejandro Mena, Michael E. von Fricken, Benjamin D. Anderson

**Affiliations:** 1 Department of Environmental and Global Health, College of Public Health and Health Professions, University of Florida, Gainesville, FL 32610, USA; amenasanchez@ufl.edu (A.M.); mvonf@phhp.ufl.edu (M.E.v.F.); 2 Emerging Pathogens Institute, University of Florida, Gainesville, FL 32610, USA

**Keywords:** highly pathogenic avian influenza, H5N1, United States

## Abstract

Highly Pathogenic Avian Influenza H5N1 (HPAI H5N1) was first detected in chickens in Scottland in 1959 and has since circulated globally, causing regular outbreaks among different animal species, as well as incidental infections in humans. In this scoping review, the epidemiology and impact of HPAI H5N1 among migratory birds, poultry, and cattle in the United States were analyzed, with a particular focus on outbreaks since January 2022. Following PRISMA guidelines, a total of 27 articles were identified for this review. Publicly available data and reports from the USDA and CDC were also evaluated and summarized. The identified articles primarily included epidemiological studies of detections in wild birds, mammals, and case reports on H5N1 and transmission dynamics among cattle, with a notable absence of poultry-focused reports. Wild birds, especially migratory species, have played an important role in virus dissemination. Studies among mammals, including seals, bears, and domestic cats, along with the emerging outbreak among cattle, highlight the virus’s ability to adapt to diverse hosts, with the possibility of mammal-to-mammal transmission. Despite the low number of human infections, the zoonotic risk of the disease and the possibility of a human outbreak remain significant. The complexity and risks associated with the virus, in comparison with the limited current scientific studies in the United States, demand further investigations to mitigate its impact on animals, ecosystems, and human health.

## 1. Introduction

The first detection of H5N1 highly pathogenic avian influenza virus (HPAI H5N1) (A/chicken/Scotland/59) occurred in chickens in Scotland in 1959 [1]. HPAI H5N1 A/turkey/England/50-92/91 was then identified as the cause of outbreaks among turkeys in England in 1991 [1]. In 1996, Goose/Guangdong/1/96 (Gs/Gd/1/96) and Goose/Guangdong/2/96 (Gs/Gd/2/96) were detected in geese being farmed in Southern China [2]. It is this lineage that then affected live poultry markets in Hong Kong in 1997 and was responsible for 18 human infections and six deaths [3]. Over time, HPAI H5N1 has continued to evolve and adapt to multiple hosts [2,4] and has caused more than 800 cases of human infection globally between January 2003 and December 2023, with a case fatality rate (CFR) of 56% [5]. Beginning in January 2022, the current clade (HPAI-H5N1 2.3.4.4b) has caused major outbreaks in the United States among birds, mammals, dairy cattle, and humans.

In this review, the epidemiological history of HPAI H5N1 in the United States is summarized, using scientific literature and official agency reports, to provide a better understanding of the disease, its impacts, risks, gaps, and key considerations that apply to both previous detections and the current outbreak.

## 2. Materials and Methods

### 2.1. Search Strategy and Selection Criteria

The search strategy and selection criteria were aligned with the Preferred Reporting Items for Systematic Reviews and Meta-Analyses (PRISMA) guidelines, which provide a standardized framework to ensure transparency and rigor in systematic reviews [6]. A systematic online search was conducted in three scientific abstract indexing databases (PubMed, Web of Science, and CAB Abstracts) with no restriction on the year of publication, performed on 28 October 2024, using the following structured search query: ((Avian Influenza OR Highly Pathogenic Avian Influenza OR HPAI OR H5N1) AND (Outbreak OR cases OR prevalence OR epidemiology) AND (USA OR US OR United States OR United States of America)). On the same date, another search query focused on cattle was conducted using the following structured search query: H5N1 AND Cattle. Subsequently, a manual review and selection process was performed based on the inclusion and exclusion criteria.

PROSPERO does not accept scoping reviews, literature reviews, or mapping reviews. As a result, this manuscript has not been registered with PROSPERO.

### 2.2. Inclusion and Exclusion Criteria

The inclusion and exclusion criteria encompassed published scientific journal entries that were epidemiological investigations or clinical case-outbreak reports conducted on animals or humans in the United States. Reviews, commentaries, perspectives, personal opinions, and diagnostic assay evaluations were excluded.

### 2.3. Inclusion of Gray Literature

Due to the limited scientific literature regarding HPAI H5N1, and in response to the ongoing outbreak of H5N1 in the United States, particularly among dairy cattle, data and reports from the United Sates Department of Agriculture (USDA) and the United States Centers for Disease Control and Prevention (CDC) were also included, covering the period from the start of the outbreak in January 2022 to the end of November 2024.

## 3. Results

### 3.1. Search Results and Study Selection

**Figure 1:**
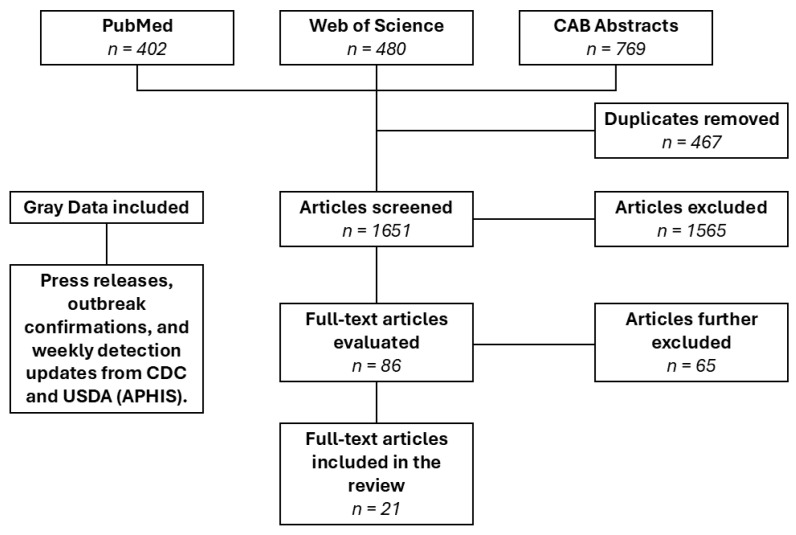
PRISMA flow diagram of general HPAI H5N1 study selection process for systematic review.

For the primary search query, 2118 articles were obtained from the databases. After removing duplicates, 1651 articles (769 from CAB Abstracts, 402 from PubMed, and 480 from Web of Science) underwent title and abstract review, resulting in 86 articles. After full-text review, 65 articles were excluded, leaving 21 articles (Figure 1). Of these 21 articles, 6 (28.57%) were studies conducted before 2016, of which 4 (19.04%) included positive detections of HPAI H5N1 in wild birds. There was a clear absence of articles about HPAI H5N1 in the United States between 2016 and 2022, with 16 articles (76.19%) published after 2022.

**Figure 2:**
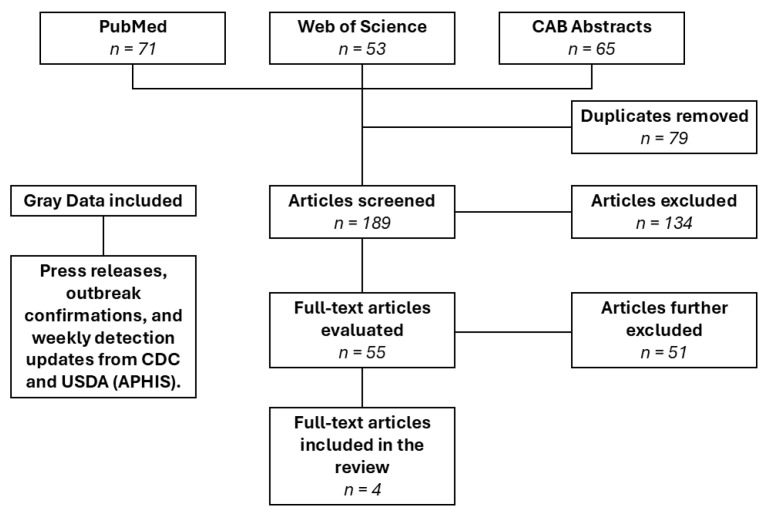
PRISMA flow diagram of cattle study selection process for systematic review.

For the secondary search query focused on cattle, 268 articles were obtained from the databases. After removing duplicates, 189 articles (65 from CAB Abstracts, 71 from PubMed, and 53 from Web of Science) underwent title and abstract review, resulting in 55 articles. After full-text review, 51 articles were excluded, leaving 4 articles (Figure 2).

From the gray literature sources, all reports and confirmations of HPAI H5N1 from the USDA and CDC beginning in 2022 through 28 October 2024, among backyard and commercial poultry, wild birds, mammals, dairy cattle, and human cases, were considered. These data were gathered, compared, and summarized for this review.

### 3.2. Epidemiology of HPAI H5N1 in the US Prior to 2022

According to the CDC, the current outbreak of HPAI H5N1 that began in early 2022 marked the first detection of the virus subtype since 2016 [7]. This is also reflected in technical reports of previous detections in the country, where other strains of avian influenza viruses (AIV) caused major outbreaks. According to the USDA, the outbreak that occurred from 2014 to 2015 was caused by H5Nx viruses from clade 2.3.4.4 of H5 but not N1. The main subtype responsible was H5N2, with only four detections of H5N1 in one county [8]. The last significant outbreak of AIV before the current one was caused by H7N8 in 2016 and occurred in Indiana, where both HPAI and low pathogenic avian influenza (LPAI) were present [9].

Scientific papers prior to 2022 reported H5N1 strains that were identified only as ‘detection’ events, and were not associated with outbreaks or case reports. A descriptive study conducted by E. Diskin et al. analyzed 77,969 samples from multiple species of waterfowl collected from 1976 to 2015 across the United States, identifying HA/H1-16 and all 9 NA subtypes with 96 (HA-NA) combinations, yielding a total of 8427 AIV isolates and 18 (0.023% of the total, and 0.21% of positive AIV samples) detections of H5N1 [10]. The subtypes H3N8, H4N6, and H6N2 were the most common in this study (22%, 20%, and 11%, respectively).

Another study conducted by Hollander et al. from 2007 to 2016 in Minnesota analyzed 13,228 samples from waterfowl, primarily mallards (n = 9133, 69.04%). Of these samples, 2329 were positive for AIV, and of those, HA and NA subtypes were identified in 2106 samples. Among these, 11 (0.083% of the total and 0.47% of AIV detections) were positive for H5N1 [11]. The most prevalent HA subtypes during this period were H3 and H4 (65.7%), with H3N8 and H4N6 being the most predominant.

Zowalaty et al. collected 7260 samples from waterfowl between June and September 2008 across multiple locations in the United States. In this study, 890 (12.3%) isolates tested positive for AIV, with 148 (2.04%) samples positive for AIV H5, and 16 (0.22% of total samples, 1.80% of AIV-positive samples, and 10.81% of H5 samples) positive specifically for the LPAI H5N1 strain [12]. Despite not being HPAI, these results are included because all H5N1 strains were detected in a mixture with other H5Nx subtypes, highlighting the potential for mutation into a highly pathogenic form.

In a study conducted by V. Goekjian et al., sampling was conducted from two different locations (winter and summer) from 2004 to 2006 in North Carolina. A total of 2889 samples were collected from various species of waterfowl, of which 1 (0.0035%) tested positive for H5N1 [13]. Notably, this H5N1 detection was combined with H4N1 in an American black duck.

Dusek et al. analyzed 1231 samples collected from December 2014 to January 2015 in Oregon and Washington from hunter-harvested wild bird species. Of these, 172 (14%) were positive for AIV, and 21 (12.2%) were positive for H5 subtypes. Eleven samples were characterized, and of these, 3 (0.24% of total samples, 14.3% of H5 subtypes, and 27.3% of characterized samples) were positive for H5N1 [14]. These samples were collected from healthy hunted birds, indicating their potential to spread the virus widely. Additionally, H5N2 and H5N8 were the most detected strains.

Last, the study conducted by C. Arriola et al. among humans exposed to infected birds was included despite finding no evidence of H5N1 or other AIV infection. The study actively tested 164 individuals who self-reported exposure to poultry infected with HPAI H5NX. No infections were found among these individuals, indicating a low risk of transmission to humans at that time [15].

### 3.3. Epidemiology of HPAI H5N1 in the US Since 2022

#### 3.3.1. Outbreaks in Poultry

**Figure 3:**
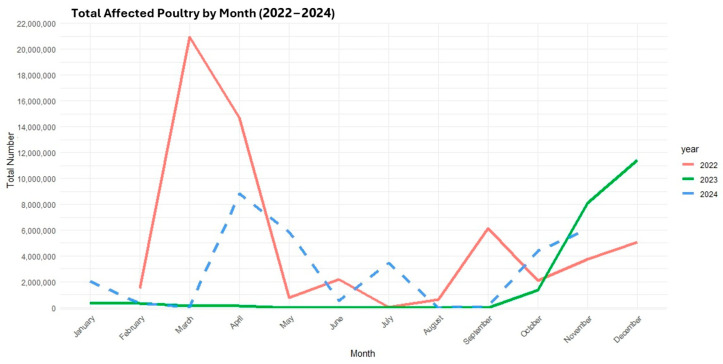
Total number of HPAI H5N1 poultry detections in the United States from January 2022 to October 2024.

There were no scientific publications identified in this review that included poultry. However, gray literature from the CDC and USDA were available. According to the CDC, as of 20 November 2024, a total of 108,412,219 infected birds in commercial and backyard poultry flocks in nearly all US states had been affected since the first detection of HPAI H5N1 clade 3.2.4.4b in early 2022 (Figure 3) [6]. The annual interim report from the USDA in 2023 compared the impact of HPAI H5N1 in post-2022 with outbreaks that had occurred in 2014–2015, showing a incidence rate of the HPAI H5N1 23 times higher than H5N2 [16].

#### 3.3.2. HPAI H5N1 Outbreaks Among Wild Birds

**Figure 4:**
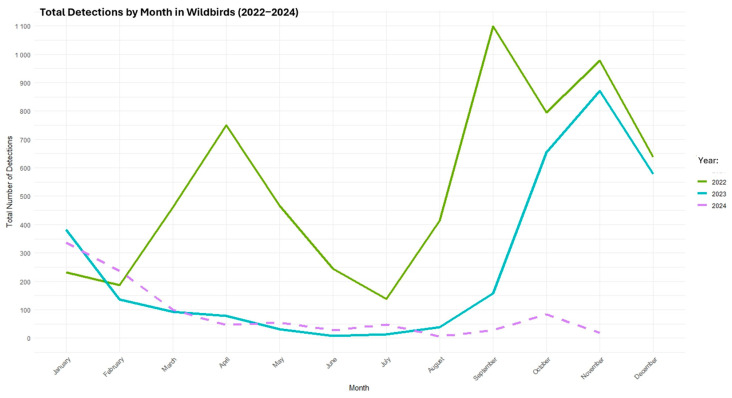
Total number of HPAI H5N1 detections among wild birds in the United States from January 2022 to October 2024.

Clade 2.3.4.4b H5N1 HPAI was reported in a wild bird sample from Colleton County, South Carolina [16]. According to the USDA, by 20 November 2024, H5N1 was detected in a total of 10,604 wild birds across the country (Figure 4) [17].

S. Teitelbaum et al. studied 43 samples from mallards in early January 2022 in Tennessee, with 11 (25.5%) testing positive for H5N1. They found that the mallards showed no clinical signs or significant changes in body composition, nor any alterations in flight patterns and routes, concluding that these species play an important role in the spread and distribution of the disease in the US [18].

M. Nemeth et al. conducted a study from January to March 2022 in the Southeastern US, evaluating 22 bald eagles postmortem, all of which tested positive for H5N1 (100%) [19]. These H5N1 detections were associated with a reduced nesting success rate in Georgia and Florida.

A study conducted on atypical wild bird species was performed between February 2022 and March 2023 by M. Ringenberg et al. A total of 9368 birds from eight orders were tested during morbidity/mortality events, with 1543 detections of AIV, resulting in an overall prevalence of 17.8%. The highest prevalence rates were found among Cathartiformes (vultures) at 53.3%, followed by Strigiformes (owls) and Accipitriformes (hawks and eagles) at 22.1% and 20.0%, respectively. These findings suggest that scavenging and predatory birds play an important role in the transmission of AIV, likely due to their consumption of infected carcasses [20].

Malmberg et al. conducted a study in April 2022 on wild turkeys found near poultry flocks positive for H5N1 in Wyoming. Of the 43 carcasses found, 11 (25.6%) samples were collected, all of which tested positive for H5N1. In addition to the comprehensive pathological analysis, they concluded that there is evidence of spillback and spillover from poultry to wild birds and vice versa [21].

Another study among wild birds was conducted by A. Krueger et al., who analyzed a case report from an urban hospital in Maine in December 2022. Two Canadian geese were found on the hospital grounds and tested positive for H5N1. This report underscores the potential risk of zoonotic virus exposure beyond just agricultural or wildlife habitats [22].

The next two studies were both conducted by The Raptor Center at the University of Minnesota, which tracks HPAI outbreaks in wild birds. The first study, conducted by V. Hall et al., described the results of HPAI surveillance from March to December 2022. Out of 996 birds admitted, 213 (21.4%) tested positive for HPAI, spanning 12 species, with great horned owls, red-tailed hawks, and bald eagles being the most affected. Of the 213 positive cases, 133 (62%) were alive at the time of admission, while 80 (38%) were dead on arrival. Among the live birds, 83% showed clinical signs, such as neurological symptoms [23].

The second study by Wünschmann et al. (2024) focused on pathological findings. The study examined 23 raptors (6 bald eagles, 9 red-tailed hawks, and 8 great horned owls), all naturally infected with H5N1. All birds showed severe neurological signs of illness, such as seizures and unresponsiveness, leading to euthanasia in all but one case. Histological lesions were identified in multiple organs, with the brain, heart, and pancreas being most affected. Viral antigen was detected in key organs, including the brain, heart, and adrenal glands, with the owls showing the most severe and widespread infections compared to the hawks and eagles. The study concluded that scavenging and predatory behaviors likely contributed to the high infection rates among raptors, as they are exposed to infected prey or carcasses [24].

Additionally, Haman et al. (2023) conducted a study in July 2023 focusing on an H5N1 outbreak among Caspian terns and glaucous-winged gulls on Rat Island, Washington, USA. The outbreak led to significant mortality in Caspian terns, with over 1101 adult terns and 520 chicks impacted, representing approximately a 56% loss of the adult population at the breeding colony. It was estimated that between 10 and 14% of the Pacific Flyway population of Caspian terns was lost during this event, highlighting and perpetuating a declining trend since 2008. A total of 1621 birds from both species were tested, with 1543 confirmed positive for H5N1, resulting in an overall prevalence rate of 95.2% [25].

#### 3.3.3. Outbreaks in Mammals

Many outbreaks among mammals have been reported globally during the current panzootic, with cases documented in 26 countries and indications of possible mammal-to-mammal transmission in some instances [26].

According to the USDA, in the U.S., by 20 November 2024, a total of 404 mammal cases had been reported during this outbreak, involving diverse species such as foxes, seals, and raccoons, among others [27]. A summary of these reports by species is provided (Table 1).

These findings were also confirmed in a study conducted by J. Elsmo et al., which examined 67 wild terrestrial mammals (50 red foxes, 6 striped skunks, 4 raccoons, 2 bobcats, 2 Virginia opossums, 1 coyote, 1 fisher, and 1 gray fox), including those naturally infected with H5N1. A full genotype analysis was performed on samples collected from 48 of the animals. Additionally, a description of clinical signs of illness was provided, showing neurological abnormalities in most cases [28].

Another study was conducted by W. Puryear et al. among harbor and gray seals in Maine between June and July 2022. A total of 175 seals were found stranded on the coast, and 41 samples were collected, of which 19 (42.2%) tested positive for H5N1. These findings are particularly relevant because birds are not a typical food source for seals, suggesting that bird-to-seal spillover may have a low species barrier and that environmental transmission contributed to the outbreak. There is insufficient evidence for seal-to-seal transmission [29].

Sillman et al. conducted a case report involving three domestic cats that tested positive (100%) for H5N1 at different times throughout 2023 in Nebraska. A comprehensive description of clinical signs and pathology was provided, highlighting the presence of neurological abnormalities similar to those observed in wild mammals. All three cats were euthanized, underscoring the importance of recognizing the potential for domestic mammals to be infected with H5N1, thereby increasing the risk of zoonotic transmission to humans [30].

Stimmelmayr et al. reported the natural infection of the 2.3.4.4b clade of H5N1 in a juvenile polar bear in Alaska, underscoring the role of the hunters’ community in wildlife surveillance and the detection of emerging pathogens in the Arctic region [31].

Additionally, Haman et al. (2023) studied an outbreak affecting marine mammals in the area, with 16 harbor seals reported stranded, of which 5 (31.3%) tested positive for H5N1. These were the first documented detections of HPAI H5N1 in marine mammals on the Pacific coast of North America. The seals displayed symptoms consistent with viral infections, such as neurological abnormalities and respiratory distress, and most were found in close proximity to areas heavily populated by infected terns and gulls [25].

Finally, a study conducted by A. Murawski et al. reported the detection of H5N1 in a bottlenose dolphin in Florida, USA. The pathological analysis revealed neurological damage, with the brain containing the highest viral load, consistent with similar findings in other mammals. Genetic sequencing confirmed that the strain was part of the current circulating strain in the U.S [32].

#### 3.3.4. Outbreaks in Dairy Cattle

HPAI H5N1 was first detected in dairy cattle in Texas in March 2024 among two domestic dairy cows and two cats from the panhandle region of northern Texas, where dead wild birds were found near the farms. The reported symptoms in cattle were nonspecific, including reduced appetite, decreased milk production, and a yellowish colostrum-like color in the milk, with possible cow-to-cow transmission [33]. Genomic sequencing of the same samples highlighted no mutations indicating increased risk for human-to-human transmission, but did note tissue tropism to the mammary glands [34]. A similar outbreak of HPAI H5N1 among dairy cattle was also later reported to have occurred during the same time period (March 2024) [35]. A later study analyzed samples from nine infected farms in Texas, New Mexico, and Kansas, finding the highest viral load and positive detections in milk. This study also provided insights into the significant role of different species interactions with the detected strains and the virus’s presence in wildlife [36].

Another study by Gimenez-Lirola et al. evaluated seroconversion and viral RNA presence in serum and milk from dairy herds affected by the virus across farms in Texas, Kansas, and Michigan. The study involved 66 animals from affected herds, from which 161 serum and 103 milk samples were collected between March and April 2024. Samples were obtained from both symptomatic and asymptomatic animals at the time of sampling [36]. Viral loads were found to be highest in milk during the acute phase of infection, with 16 out of 19 milk samples from symptomatic cows testing positive via RT-qPCR at week 0 (initial clinical onset), decreasing to 11 out of 19 at week 2, and no viral RNA detected by week 4. The serological analysis revealed that while PCR testing became less effective after the first two weeks, ELISA testing detected antibodies in 96% of serum samples and 89% of milk samples by week 4 [37].

**Figure 5:**
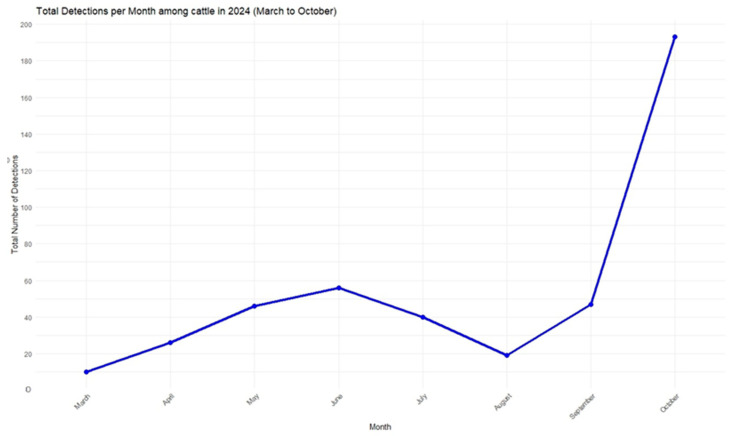
Total number of HPAI H5N1 detections represented in blue line among dairy cattle herds in the United States from March to October 2024.

On 29 March 2024, the USDA confirmed the detection of H5N1 in dairy cattle in Texas, Kansas, and Michigan, followed by subsequent confirmations in herds in New Mexico and Idaho [38]. As of 20 November 2024, these states, along with South Dakota, Colorado, Wyoming, Minnesota, Iowa, Oklahoma, Ohio, North Carolina, Utah, and California, had confirmed cases of HPAI in dairy herds. California alone reported 398 cases as of that date. In total, 612 dairy herds across 15 states were affected (Figure 5) [39].

According to USDA recommendations for state animal health officials, accredited veterinarians, and producers, the primary signs in cattle that warrant suspicion and testing for H5N1 are broad and overlap with many other diseases. These symptoms include decreased milk production, reduced appetite, thickened or discolored milk, lethargy, fever, and dehydration, suggesting that undiagnosed cases may exist in other states that have not tested sick cattle for the virus [40].

Due to the novel reassortment of the virus, which has also resulted in infections in goats and alpacas [41], the full impact of H5N1 among ungulates and other stabled animals remains unknown, with government agencies acknowledging that they are still gathering information [40,42].

It has been established that H5N1 is present in the milk of infected cows, and a challenge study in mice orally inoculated with raw milk containing HPAI H5N1 showed that the animals developed signs of illness beginning within 24 h of inoculation, with high viral titers in the respiratory organs and moderate titers in other organs such as the brain, spleen, and mammary glands. These were similar to findings in dairy cows even when the mice were not lactating [43]. These findings led to public recommendations to avoid raw milk consumption and emphasized the importance of pasteurization. Notably, even with positive RNA detections of the virus in pasteurized commercial milk, the virus itself is not viable or infectious [44]. Additionally, studies demonstrate that H5N1 has a high affinity for sialic acid receptors in the respiratory tract, with a particular concentration in the mammary glands of dairy cattle [45].

To reduce the impact of the disease among cattle as well as the risk to humans, the USDA has restricted cattle movement, requiring a negative test for influenza A virus from an approved National Animal Health Laboratory Network (NAHLN) laboratory. Any interstate movement must comply with conditions specified by USDA APHIS [46]. Additionally, the USDA began to promote best practices among farm owners and workers, including the use of personal protective equipment (PPE), implementation of biosecurity measures, ensuring veterinary care, conducting H5N1 testing, and managing sample shipping. The USDA also began compensating cattle producers for positive detections to mitigate the economic impact [47].

The USDA has also implemented the Dairy Herd Status Program to monitor and manage herd health. This program mandates regular surveillance and testing for H5N1, conducted through approved National Animal Health Laboratory Network (NAHLN) facilities. Herds that meet stringent testing requirements and adhere to biosecurity protocols are granted a clear status, enabling them to continue operations and transport livestock without restrictions [48].

In terms of risk and exposure, a study documented 11 wild waterfowl in or near eight commercial livestock facilities in Washington and California using GPS telemetry data. The study showed that wild ducks utilized dairy and beef cattle feedlots and facility retention ponds both day and night, suggesting these areas were used for roosting and foraging. This behavior demonstrates the connectivity between natural reservoirs of HPAI and livestock [49].

#### 3.3.5. Outbreak in Humans

From the databases, notes on in-field health monitoring, testing, and case identification for individuals exposed to H5N1 in Michigan (where the second and third detections in humans were identified), along with a report of a cluster of human infections in Colorado and serological testing are available. These notes describe the local surveillance efforts and the clinical findings of positive cases, highlighting exposure through contact with raw milk, which led to conjunctival samples testing positive for H5N1. No masks or respirators were used by the two positive individuals and no clinical symptoms were observed [50].

Additionally, there is a report of a cluster of human infections with influenza A(H5) associated with poultry exposure, documented in July 2024 at two poultry facilities in Colorado. Out of 663 workers screened, 109 (16.4%) reported symptoms, and 9 (8.3%) tested positive for H5N1. All positive cases exhibited mild symptoms, with conjunctivitis being the most common. The affected workers received oseltamivir for treatment, and no severe complications were observed. The report emphasized the importance of PPE, noting that compliance varied between the two facilities. Despite high PPE use at one facility, infections were still detected, underscoring the ongoing risk of HPAI transmission to humans working closely with infected animals [51].

According to reports by the CDC, by 20 November 2024, 54 human cases of HPAI H5N1 had been confirmed in the U.S. since 2022, with one case in 2022 and the remaining 53 occurring in 2024. Of these 2024 cases, 31 were linked to exposure to dairy cattle, 21 were from contact with infected poultry, and 1 case remains of unknown origin [52].

The first human detection in 2022 occurred in Colorado and involved a worker exposed to infected poultry. This individual experienced only mild fatigue and recovered after treatment with oseltamivir. In July 2024, a cluster of nine additional cases was reported in the same state, all involving poultry workers. These workers exhibited conjunctivitis as the most common symptom, with some reporting respiratory symptoms. This number increased further with five additional detections among poultry workers in Washington by October 2024 [53].

The dairy cattle-related cases total 32, with all detections occurring in 2024. Notably, 28 dairy workers in California were affected across multiple farms, with no evidence of human-to-human transmission [54].

For the case of unknown origin, on 6 September 2024, an individual in Missouri tested positive for H5N1 but had no reported direct contact with infected poultry, dairy cattle, or any other animal species [55]. The individual was hospitalized and later fully recovered but subsequently raised concerns about the potential for unrecognized exposure routes and asymptomatic cases, as one close contact to the index case also tested positive by serological assays, confirming prior exposure to the virus [56]. Another serological study later demonstrated that 7% of dairy workers also had evidence of recent HPAI A(H5) virus infection, but with limited-to-no symptoms [57]. Another human infection of unknown origin was also detected on 13 November 2024 in British Columbia, Canada. The infection occurred in a teenager who was also hospitalized with severe symptoms. Genomic sequencing indicated that the virus was related to the ongoing outbreak in poultry in the region but the exact source of infection remains undetermined [58].

## 4. Discussion

**Figure 6:**
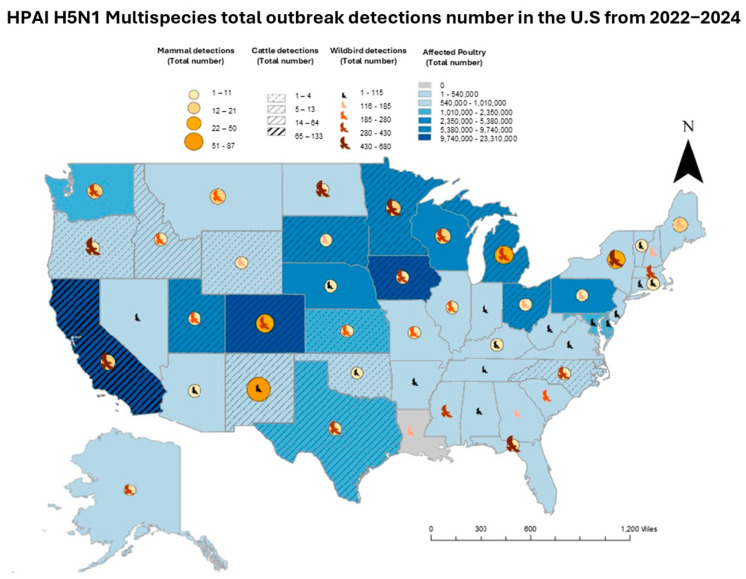
Multispecies total HPAI H5N1 outbreak detections number in the United States from 2022 to 2024.

The current outbreak in the U.S. has affected more than 100 million birds, making it the largest and most significant AIV event in the country [7]. A map showing total outbreak detections in the United States across all species groups from 2022 to 2024 is included to visualize the states with the highest number of detections and multiple species group interactions (Figure 6).

The infection among a wide range of mammalian species, including seals, a polar bear, and various terrestrial carnivores such as domestic cats, has demonstrated the high adaptability of this latest strain of HPAI H5N1 to new hosts [26,27,28,29,30]. In the case of the affected seals, a low species barrier for transmission between diverse species appears possible. Additionally, the signs and pathology are consistent among infected mammals despite their physiological and morphological differences, with neurological issues being one of the most common signs of infection [27,29]. Studies related to uncommon reservoirs of the disease demonstrate a link between exposure to infected wild birds and the presence of the disease [19,21,28,29], highlighting the importance of spillovers caused by these species. This evidence suggests that wild birds are a common source of the spread and prevalence of the disease. In many cases, wild birds show no signs of illness or changes in behavior, health status, or flight patterns [14,18], allowing them to disseminate the virus along their migratory routes both nationally and internationally [49]. The high prevalence of the disease in the U.S. poses a significant risk not only to poultry and mammals but also to wild bird populations, which play a crucial role in ecosystems [19]. Many studies have emphasized the importance of efforts to prevent exposure to infected animals due to the evident adaptability of the virus and its potential to cause human infections [15,22,30].

Despite many sporadic cases being reported globally (882 cases of human infection from 1 January 2003, to 21 December 2023, across 23 countries) [6], only 54 cases have been confirmed in the U.S. as of the date of our search, all who recovered [53,59]. This suggests that the current risk of transmission to humans remains low. However, recent evidence of potential mammal-to-mammal transmission [26,28], cow-to-cow transmission [34] and the high prevalence and adaptability of the virus necessitate robust surveillance and prevention measures to mitigate the risk of a possible human outbreak in the future.

The information sourced from the USDA and CDC was collected through active and passive surveillance approaches, including continuous monitoring of humans with dairy cattle or poultry exposure for possible infection [16]. On 30 October 2024, the USDA and state veterinary officials confirmed the first detection of H5N1 in swine at a backyard farming operation in Oregon. The infected farm housed a mix of poultry and livestock, including swine, which shared water sources, housing, and equipment, facilitating interspecies transmission. This marked the first detection of H5N1 in swine in the United States [60], underscoring the virus’s ability to cross species barriers and the importance of surveillance across diverse animal populations.

The ongoing transmission of H5N1 among dairy cattle underscores the importance of continued monitoring and testing among dairy cattle and poultry settings. Wastewater testing has also been an effective tool for detecting broader outbreaks of H5N1 in various states [61]. Between May and July 2024, 203 wastewater sites across 41 states were tested for the H5 subtype, and 24 sites in nine states confirmed detections of H5 viral RNA. Some of these sites had potential sources from animal facilities, including eight milk-processing plants identified as contributors to H5 detections in wastewater, aligning with known outbreaks in dairy cattle herds [62].

Another example of environmental sampling is the work of Singh et al., who collected environmental samples in Kansas. The study involved 11 environmental swab samples taken from surfaces, including rubber, plastic, and concrete at an infected dairy farm. After testing, 10 of the 11 samples were positive, with the highest viral loads found on rubber surfaces [63]. Furthermore, sequencing analysis revealed mutations closely related to strains isolated from human cases, underscoring the potential zoonotic transmission risks and the possibility of human outbreaks [63]. This demonstrates that environmental sampling can be an effective tool for monitoring and characterizing viral spread within farms.

Despite human infections being limited and the risk of human-to-human transmission deemed low by the USDA and CDC, the potential for the virus to continue mutating and or reassorting remains high. This means that future risk scenarios are more uncertain and could change rapidly. One study has shown that H5N1 isolated from an infected dairy worker (who only had conjunctivitis as a symptom) was lethal in inoculated ferrets and was transmissible via the aerosol route between separated ferrets [64]. Another study found similar results in ferrets through direct and indirect contact, including fomites and airborne transmission [65]. Ferrets are a gold standard for modeling influenza virus infection and for testing vaccines and therapeutics. These findings suggest biological plausibility that H5N1 could potentially bind and replicate in human respiratory tract cells, raising the concern for potential human-to-human transmission.

Last, the limited presence of scientific studies in the context of the recent outbreak may be attributed to the challenges of working with HPAI H5N1, which requires access to a Biosafety Level 3 facility. Additionally, even though the USDA provided an exemption for the Select Agent classification for HPAI H5N1, there are some institutions that may still be hesitant to work with HPAI H5N1 samples should that exemption be rescinded. There are also challenges in conducting surveillance in commercial dairy and poultry farms due to the economic risks associated with H5N1 detections. The USDA indemnity program has sought to mitigate this risk, but may not be adequate enough in compensation to overcome the economic barriers. Other factors, such as consumer sentiment, may also play a role.

## 5. Conclusions

In conclusion, while the current outbreak of HPAI H5N1 in the United States has primarily affected birds, its spread to diverse mammalian hosts, including dairy cattle, highlights the virus’s adaptability and the ongoing risk of cross-species transmission. Despite the low number of human cases, the potential for undiagnosed asymptomatic human infections raises concerns about the true prevalence of the virus among exposed populations. Furthermore, evidence of mammal-to-mammal transmission, the identification of H5N1 in swine, and the recent detection of a severe case of H5N1 in a teenager underscore the increasing risk of viral mutations that could enhance human transmissibility. Future research should prioritize expanding surveillance in both animal and human populations, including environmental sampling techniques; investigating the mechanisms behind cross-species adaptation, particularly in mammals; and enhancing biosecurity and testing measures to minimize zoonotic spillover. Additionally, particular attention should be given to addressing the exposure risks among dairy and poultry farm workers, as they represent the most at-risk populations due to their direct and prolonged contact with susceptible animals. Proactive measures in these areas are essential to mitigate the impact on animal and human health, prevent any potential future outbreaks, and ensure that protective measures reach the most vulnerable workers in this scenario. International collaboration will be critical to sharing data and insights, given the global nature of migratory patterns and interspecies contact, which continue to drive the spread of this pathogen.

## Figures and Tables

**Table 1:** Total number of HPAI H5N1 detections by species in mammals in the United States from 2022 to October 2024.

Species Detection by Year	2022 (N = 110)	2023 (N = 113)	2024 (N = 181)	Overall (N = 404)
Abert’s squirrel	0 (0%)	1 (0.9%)	0 (0%)	1 (0.2%)
American black bear	1 (0.9%)	3 (2.7%)	0 (0%)	4 (1.0%)
American marten	0 (0%)	1 (0.9%)	0 (0%)	1 (0.2%)
American mink	0 (0%)	0 (0%)	1 (0.6%)	1 (0.2%)
Amur Leopard	1 (0.9%)	0 (0%)	0 (0%)	1 (0.2%)
Amur tiger	0 (0%)	1 (0.9%)	0 (0%)	1 (0.2%)
Bobcat	2 (1.8%)	4 (3.5%)	3 (1.7%)	9 (2.2%)
Bottlenose dolphin	1 (0.9%)	0 (0%)	3 (1.7%)	4 (1.0%)
Coyote	1 (0.9%)	0 (0%)	0 (0%)	1 (0.2%)
Deer mouse	0 (0%)	0 (0%)	14 (7.7%)	14 (3.5%)
Desert cottontail	0 (0%)	0 (0%)	1 (0.6%)	1 (0.2%)
Domestic cat	1 (0.9%)	12 (10.6%)	40 (22.1%)	53 (13.1%)
Fisher	1 (0.9%)	2 (1.8%)	0 (0%)	3 (0.7%)
Grey seal	1 (0.9%)	0 (0%)	0 (0%)	1 (0.2%)
Grizzly bear	3 (2.7%)	0 (0%)	0 (0%)	3 (0.7%)
Harbor seal	16 (14.5%)	5 (4.4%)	0 (0%)	21 (5.2%)
House mouse	0 (0%)	0 (0%)	82 (45.3%)	82 (20.3%)
Kodiak bear	1 (0.9%)	0 (0%)	0 (0%)	1 (0.2%)
Mountain lion	0 (0%)	22 (19.5%)	1 (0.6%)	23 (5.7%)
North American river otter	0 (0%)	1 (0.9%)	0 (0%)	1 (0.2%)
Polar bear	0 (0%)	1 (0.9%)	0 (0%)	1 (0.2%)
Praire vole	0 (0%)	0 (0%)	1 (0.6%)	1 (0.2%)
Raccoon	7 (6.4%)	8 (7.1%)	7 (3.9%)	22 (5.4%)
Red fox	54 (49.1%)	31 (27.4%)	14 (7.7%)	99 (24.5%)
Skunk (unidentified)	6 (5.5%)	0 (0%)	1 (0.6%)	7 (1.7%)
Striped skunk	11 (10.0%)	20 (17.7%)	11 (6.1%)	42 (10.4%)
Virginia opossum	3 (2.7%)	1 (0.9%)	2 (1.1%)	6 (1.5%)

## Data Availability

Data available in references and Supplementary Materials [66]. All data supporting the reported results are publicly available and can be downloaded from the CDC and USDA databases. Further details on accessing these datasets can be found directly on the respective organizations’ websites.

## Supplementary Materials

The following supporting information can be downloaded at: https://www.mdpi.com/article/10.3390/v17030307/s1, Table S1: Preferred Reporting Items for Systematic reviews and Meta-Analyses extension for Scoping Reviews (PRISMA-ScR) Checklist.
